# **Artificial intelligence accelerates the identification of nature-derived potent LOXL2 inhibitors**

**DOI:** 10.1038/s41598-025-95530-9

**Published:** 2025-03-27

**Authors:** Xiaowei Jia, Meng Liu, Yushi Tang, Jingyan Meng, Ruolin Fang, Xiting Wang, Cheng Li

**Affiliations:** 1 School of Traditional Chinese Medicine, Tianjin University of Traditional Chinese Medicine, Tianjin, China; 2 Sijiqing Hospital, Beijing, China; 3 School of Traditional Chinese Medicine, Beijing University of Chinese Medicine, No.11 Bei San Huan Dong Lu, Beijing, 100029 China; 4 Tian Jin Key Laboratory of Modern Chinese Medicine Theory of Innovation and Application, No.10 Poyang Lake Road, Tianjin, 301617 China

**Keywords:** LOXL2, Cancer, Deep learning, Drug discovery, Drug discovery, Cancer, Cancer therapy

## Abstract

The role of LOXL2 in cancer has been widely demonstrated, but current therapies targeting LOXL2 are not yet fully developed. We believe that selective nature-derived inhibition of LOXL2 may provide a better therapeutic approach for the treatment of cancer. Therefore, we adopted a comprehensive approach combining deep learning and traditional computer-aided drug design methods to screen LOXL2 selective inhibitors. Bioactivity and affinity of the potential LOXL2 inhibitors were determined by molecular docking and virtual screening. At the same time, we experimentally tested the effect of potential LOXL2 inhibitors on cancer cells. Validation showed that it could inhibit proliferation and migration, promote apoptosis of CT26 cells, and reduce the expression level of LOXL2 protein. As a result, we identified a potent LOXL2 inhibitor: the natural product Forsythoside A, and demonstrated that Forsythoside A has an inhibitory effect on tumors.

**Supplementary Information:**

The online version contains supplementary material available at 10.1038/s41598-025-95530-9.

## Introduction

The lysyl oxidase (LOX) family, which includes the founding member LOX and four LOX-like enzymes (LOXL1 − 4), was originally identified as a secreted enzyme capable of catalyzing the oxidation of peptidyl-lysine. This process results in the formation of covalent crosslinks between collagen and elastin monomers^1^. Research has demonstrated that LOXL2 is present in the cytoplasm, nucleus, and microenvironment, and it is involved in a wide range of cellular processes associated with cancer^2^. LOXL2 is abnormally overexpressed in patients with numerous cancers, could promote the proliferation, migration, invasion, and metastasis of cancer cells, and is associated with poor prognosis^3^. Furthermore, LOXL2 mediates the crosstalk between cancer cells and cancer-associated fibroblasts, facilitating cancer metastasis^4^.

Given the prominent role of LOXL2 in many types of cancer, selective inhibitors of LOXL2 may turn out to be useful therapeutic agents for the treatment and have distinct advantages for their long-term therapeutic management^1,5^. However, the first attempt to development of a humanized version of the LOXL2-targeting antibody, simtuzumab, failed to show clinical effectiveness in several clinical trials that targeted several types of cancer^1^. One plausible explanation for this failure is the antibody was too specific, not internalized effectively, and lastly failed to inhibit LOXL2 intracellular activities.Given these studies, attempts are currently being made that aim to develop agents that will address at least some of these obstacles. However, novel methodologies are essential to accelerate the discovery of new LOXL2 inhibitors and to reduce the financial burden associated with early lead identification. Inspired by the advancements of machine learning (ML), particularly in the realm of deep learning (DL), significant strides have been made in the fields of computational biochemistry and drugs discoverier^6^. Notably, Alex et al. utilized a deep generative model to uncover potent inhibitors of discoidin domain receptor 1 (DDR1), a kinase target implicated in fibrosis and other ailments^7^. Li et al. introduced a multitask model for simultaneous inhibition prediction of five primary CYP450 isoforms^8^. Furthermore, Li et al. identified Antifebrile Dichroa, Holarrhena antidysenterica, and Gelsemium sempervirens as potential Traditional Chinese Medicines (TCMs) for two longevity-related targets: the insulin-like growth factor 1 receptor and the insulin recepto^9^.

Studies have also demonstrated that the integration of deep learning in early drug discovery enables the exploration of vast chemical spaces, surpassing the capabilities of current experimental methodologies^10^. Nevertheless, in the context of drug discovery, the application of machine learning methods remains constrained by several factors:1) the suboptimal interpretability of models, often due to limitations in molecular representation techniques; and 2) a scarcity of validation procedures whether through diverse algorithmic approaches or biological experimental verification.

In the realm of machine learning (ML) and deep learning (DL), molecules are typically represented by fingerprint vectors that indicate the presence or absence of functional groups within the molecule, or by descriptors encompassing computable molecular properties, which necessitate expert knowledge for their construction^11,12^. However, these conventional methods fail to effectively capture the multidimensional relationships between atoms in molecules. Recently, the molecular graph has emerged as a leading-edge representation method for molecules, complemented by advancements in corresponding DL algorithms, such as graph neural networks (GNNs) and graph convolutional networks (GCNs). Choi’s research demonstrated that graph-theoretical methodologies could decipher the morphological attributes of large aggregates in various aqueous salt, osmolyte, and sugar solutions^13^. Evans’ study revealed that spatial Graph Convolutions achieve cutting-edge performance in affinity prediction^14^. Jaechang and colleagues introduced an innovative GNN-based approach for predicting drug-target interactions, which directly integrates 3D structural data, outperforming docking and other deep learning techniques in virtual screening and pose prediction^15^. Such sophisticated designs yield molecular representations that are finely tuned to the desired attribute, thereby enhancing the accuracy of property predictions compared to manually crafted representations^16^.

Although deep learning demonstrates superior feature extraction capabilities, traditional approaches in computer-aided drug design (CADD) continue to serve complementary and verification roles, given the complex nature and processes involved in drug discovery. Over the past decade, molecular docking methods have been successfully applied to numerous leading drug discoveries^17^. These methods facilitate drug design, particularly in aspects related to ligand and receptor structures and binding energies. Concurrently, biological experimentation remains essential for verification, as limited validation procedures have been applied to virtual drug screening studies. Consequently, we propose that a novel comprehensive approach may expedite the identification of nature product-derived potent LOXL2 inhibitors through the integration of deep learning algorithms, traditional CADD approaches, and biological experimental verification.

In our research, we employed integrated methodologies that amalgamated deep learning with traditional computer-aided drug design techniques, followed by experimental validation, to identify potent LOXL2 inhibitors. We screened an expansive natural product database and utilized an adjacency matrix-based molecular graph to represent molecules, employing a graph convolutional network to construct a predictive model. Subsequent to the development of this virtual screening model, we identified various novel potently active natural compounds that target LOXL2. Thereafter, we harnessed a molecular docking approach to further corroborate the active natural ligands and elucidate the structural and binding milieu based on the pivotal interactions exhibited by known inhibitors. This is followed by a comprehensive series of biological experiments. Our findings indicated that Forsythoside A novel LOXL2 inhibitor. Moreover, deep learning facilitates expedited development and identification of natural drugs, offering an innovative paradigm in nature-derived drug discovery protocols.

## Materials and methods

### Data collecting and processing

Firstly, the positive data set was generated, based on LOXL2 inhibitor and cancer inhibitor data. The LOXL2 inhibitor data was collected via searching PubMed database (https://pubmed.ncbi.nlm.nih.gov/), while cancer inhibitor data were obtained from the “Cancer-Inhibitors-Modulators” library (MCE, http://www.MedChemExpress.cn). The negative data set was generated from a comprehensive dataset^18^, which was commonly used as data set for chemical informatics research. Both positive and negative data were split into training sets and testing sets.

Subsequently, a large scale database of traditional Chinese Medicine ingredient (TCMID database, http://www.megabionet.org/tcmid/)^19^ was collected for screening, which included over 40,000 ingredients. After removing duplicates and screening based on drug-likeness, a total of 25,918 compounds were included as candidate compounds. Meanwhile, a precise and miniature natural products dataset (www.biopurify.cn) was also obtained, which included 2,042 instances and was commonly used in pharmacological research.

Subsequently, we developed an algorithm for the molecular graph representation of TCM compounds, encoding the SMILES representation of compound molecules into higher-order matrices based on graph theory. Using RDKit (http://www.rdkit.org/), we converted the SMILES format of molecular representations into Mol file format. With RDKit, we computed the adjacency matrices of the molecules, extracting atom types and graph representations of the molecules. The Pubchem^20^ CID and molecule name of these ingredients were retained for obtaining the molecule SDF file from Pubchem website (https://pubchem.ncbi.nlm.nih.gov/).

### Deep learning model construction and evaluation

The DGL-LifeSci framework (https://github.com/awslabs/dgl-lifesci) and PyTorch were utilized to construct graph neural network algorithms. The propagation rule for the graph convolutional layer is defined as:

$${\text{H}}^{{{\text{l}} + 1}} = \upsigma \left( {\tilde{\text{D}}^{{ - \frac{1}{2}}} \tilde{\text{A}}\tilde{\text{D}}^{{ - \frac{1}{2}}} \text{H}^{\text{l}} \text{W}^{\text{l}} } \right),$$

where (1) $\tilde{\text{A}} = \text{A} + \text{I}$ is the adjacency matrix of the molecule with added self-loops; (2). I is the identity matrix; (3) $\tilde{\text{D}}$ is the diagonal node degree matrix corresponding to $\tilde{\text{A}}$; (4). W^1^ is the weight matrix for the l-th neural network layer; (5). H^1^ is the output of the l-th graph network layer (also denoted as X); (6) $\upsigma ()$ is a nonlinear activation function, typically set as the ReLU function.

During the model training phase, the Adam optimizer was adopted for gradient descent optimization, and the CrossEntropyLoss function was used to calculate errors for binary classification tasks. To mitigate model overfitting and enhance robustness, hyperparameter optimization strategies such as batch normalization layers and neuronal dropout methods (with a dropout rate of 0.3) were implemented. Subsequently, the model was trained through 10,000 iterations, setting the minibatch size to 32. AUROC (area under the receiver operating characteristic curve) was chosen as the primary metrics for evaluating model performance, calculated using Numpy and Scikit-Learn (V0.23) metrics tools.

The AUROC can be interpreted as the probability that the model will rank a randomly chosen positive instance more highly than a randomly chosen negative one. In other words, it measures the ability of the model to distinguish between positive and negative classes. The formula for calculating AUC is:

$$\frac{{\sum\nolimits_{{i \in positiveClass}} {rank_{i} - \frac{{M(1 + M)}}{2}} }}{{M \times N}},$$

here (1) M and N represent the number of positive and negative samples, respectively; (2) ${\text{rank}}_{{\text{i}}}$ refers to the rank of each positive sample when all samples are sorted by their predicted probabilities of being positive; (3) The term $\frac{{{\text{M}}\left( {1 + {\text{M}}} \right)}}{2}$ is the sum of the first $\:\text{M}$ natural numbers, which would be the sum of the ranks if all positive samples were ranked at the top. It’s worth noting that AUC ranges from 0 to 1, with higher values indicating better model performance. An AUC of 0.5 means that the model has no discriminative power, while an AUC of 1 means that the model has perfect discriminative power. The optimized GNN model was designated as the DeepVS model for the virtual screening of natural products with anti-cancer activity.

### Molecular docking and molecular dynamics simulation analysis

#### Molecular docking analysis

The molecular docking simulation was conducted using the GLIDE (v7.7) module. The crystal structure of human lysyl oxidase-like 2 (hLOXL2) in a precursor state was obtained from the RCSB protein data bank (PDB ID: 5ZE3, Resolution: 2.4A°). A candidate library for molecular docking was generated based on two datasets: the natural products library and the positive prediction of a deep learning model from the TCMID database.

We prepared the ligands from the candidate library using the LigPrep module (v3.5, Schrödinger 2016-1). Next, protein preparation was performed on the PDB files of LOXL2 to ensure proper assignment of bond orders and ionization states. We then detected and defined receptor grids using the Receptor Grid Generation panel. Subsequently, molecular docking simulation was performed on the ligands and calculated binding grids of LOXL2 according to the Virtual Screening protocol. Finally, in silico ADMEproperties of the selected ligands were further analyzed. Molecular drug-likeness related indicators, such as molecular weight, aqueous solubility, human oral absorption, and liposolubility were calculated using the Ligand-based ADME/Tox Prediction panel (Maestro 11.9).

#### Molecular dynamics simulation analysis

Molecular dynamics (MD) simulations were performed using the AmberTools23 software for the preprocessing of small molecules. The RESP charge fitting was applied to the small molecules, which were modeled using the gaff molecular force field. The protein was modeled with the OPLS-AA/L force field, and the system was solvated in a cubic water box using the three-point transferable intermolecular potential (TIP3P) water model. An appropriate number of sodium ions were added to neutralize the system charge. The complex was placed in the water box, and the cutoff distances for electrostatic and van der Waals interactions were set to 1.0 nm. The time step was set to 2 fs, and long-range electrostatic interactions were corrected using the Particle Mesh Ewald (PME) method. The system temperature was maintained at 300 K, and the pressure was set to 1 bar. Two complex systems were constructed. After system setup, energy minimization was performed using the steepest descent algorithm to achieve a stable system.

Subsequently, a 100 ps NPT equilibration dynamics was conducted. Initially, the system was heated from 0 K to 300 K in an isothermal-isochoric (NVT) ensemble with constraints on the small molecule, followed by equilibration in an isothermal-isobaric (NPT) ensemble at 300 K and 1 bar. The system temperature was coupled using the V-rescale method, and the pressure was controlled using the Parrinello-Rahman method. Finally, a 100 ns MD simulation was performed on the protein-ligand complex, and the trajectory was saved for subsequent analysis.

### Biological experiment verification

#### Chemicals and materials

Forsythoside A (purity 99%) was purchased from Biopurify Phytochemicals Ltd (Chengdu, China) and dissolved in double-diluted water to prepare a stock solution. Murine CT26 cells were obtained from the cancer cell library of the Chinese Academy of Medical Sciences (ATCC CRL-2638) and were grown in RPMI 1640 medium (Pricella, Wuhan, China) with 10% fetal bovine serum (FBS) (Capricorn Scientific, Wuhan, China) and 1% penicillin-streptomycin (Gibco, Gaithersburg, USA). Cells were cultured on specific plates under conditions simulating the human body environment (5% CO_2_, 100% humidity, 37℃), and used at 75–80% confluence.

#### Cell proliferation assay

Cell Counting Kit-8 (CCK-8) (Dojindo, Kumamoto, Japan) was used to detect cell proliferation in vitro. The cells were evenly inoculated into 96-well plates (5,000 cells per well) and allowed to rest overnight for normal division and multiplication. Forsythoside A (50 µmol/L) was added to and incubated for 24 h. For the control group, a complete medium was added. Then the medium in the 96-well plate was aspirated and CCK-8 solution (10 µL) was added to each well and incubated for 1–4 h at 37 ℃. The absorbance was measured at 450 nm.

#### Wound-healing assay

Wound-healing assay was used to investigate the cell migration in vitro. The cells were inoculated into 6-well plates, and grown to 75–80% confluence. A clean break was made across the cell layer of each well. Then the wells were rinsed with PBS, incubated with Forsythoside A (50 µmol/L), and cultured for 24 h. Randomly choosing 3 visions to take photos, and analyzed the area of wound healing with ImageJ software, to quantify the cell migration.

#### Cell apoptosis assay

Annexin V-FITC/PI Apoptosis Detection Kit (Solarbio, Beijing, China) was used to detect the cell apoptosis. The cells were implanted in a 6-well plate, cultured overnight, and then treated with Forsythoside A for 24 h. According to the manufacturer’s instructions, the cells were collected using EDTA-free trypsin, resuspended with 1× AnnexinV binding solution, stained with 5 µL of Annexin V-FITC and 5 µL of PI for 15 min in the dark, and analyzed by Flow CytoMetry. Each concentration has three repeats.

#### Western blotting analysis

After being treated with Forsythoside A for 24 h, the cells were washed with PBS twice, and the proteins were harvested by RIPA lysis buffer containing PMSF(Solarbio, Beijing, China) at 4 ℃ for 15 min. The lysates were centrifuged at 12,000 g at 4 ℃ for 20 min. The protein concentration was determined by BCA protein quantitative kit (Solarbio, Beijing, China), mixed with 5× loading buffer, boiled for 10 min, subjected to electrophoresis on an SDS/PAGE gel, and transferred to PVDF membranes. After blocking with 5% skim milk for 1 h, the membranes were incubated with the primary anti-LOXL2 (1:1000, Proteintech Group, Wuhan, China) and corresponding HRP-labeled secondary antibody. Then, the proteins were visualized using an enhanced chemiluminescence solution (ECL) (Solarbio, Beijing, China). The image was captured by Bio-Rad bioimaging system, and the gray value of the membrane was quantitatively analyzed by ImageJ software (NIH, Bethesda, MD), and the corresponding β-actin (1:1000, Selleck, Shanghai, China) is standardized as an internal reference.

#### Enzyme-linked immunosorbent assay (ELISA)

LOXL2 ELISA kit was purchased from Hangzhou Zhenyoupin Biotechnology. The cells were incubated for 24 h with different treatments, then the supernatant was collected and the LOXL2 level was determined according to the manufacturer’s instructions.

### Statistics analysis and visualization

In this study, we used two powerful machine learning libraries, Scikit-Learn (v0.22.1) and Matplotlib (v3.1.2), for data reduction and visual analysis. We use the T-distributed random neighbor embedding (t-SNE) method, which is an algorithm that performs well in dimensionality reduction of nonlinear data. With this approach, we can better understand the underlying structure in high-dimensional data.

In the process of data processing, we use the Pandas software package to calculate the atomic correlation matrix. Pandas is a powerful data processing library that makes it easy to process and analyze complex data structures. In addition, the Seaborn software package (version 0.10.0) was used for data visualization for aesthetics and ease of understanding.

The experimental data is expressed in the form of mean ± standard deviation, which can more intuitively show the change in the data. In terms of statistical analysis, SPSS (version: 20.0) software was used for all relevant calculations. To compare the differences between different groups, we used Dunnett test for one-way ANOVA. This test can effectively assess whether the differences between multiple sets of data are statistically significant. In this study, we set a *P*-value of less than 0.05 as the threshold for statistical significance. With the above methods, we can analyze and compare experimental data more accurately to reveal potential differences between different groups.

## Results and discussion

### Deep learning model performance and parameter optimization

**Fig. 1:**
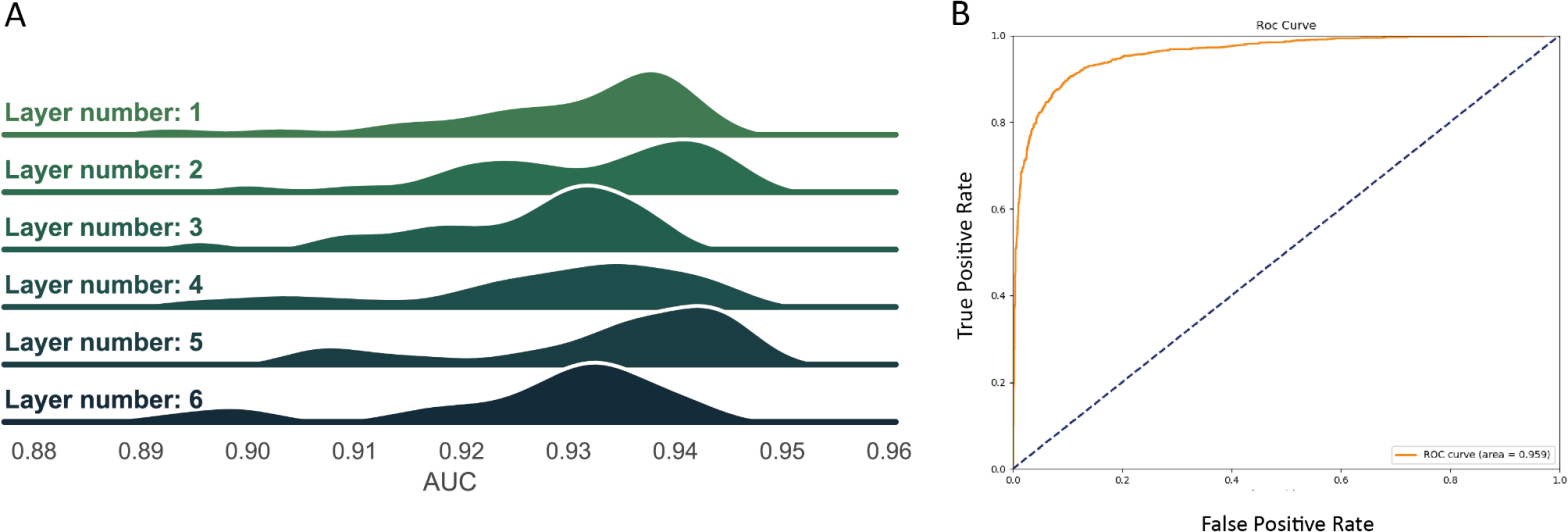
AUC metrics and layer selection for varying GCN layers: (**A**) Relationship between GCN layers and model AUC; (**B**) ROC curve of the model when three GCN layers are selected.

We enhanced a deep learning framework by employing a graph convolution network (GCN) approach. This involved the calculation of the adjacency matrix and initialization of the embedded features of the molecule to serve as model inputs. During the GCN model training phase, adjustments were made to the convolutional layer parameters to generate an optimal model. It was observed that alterations in the number of convolutional layers had a minimal impact on AUC values (as depicted in Fig. 1A), leading to the final decision to utilize two hidden layers. The GCN model achieved an impressive mean AUC score of 0.942 in classification prediction tasks (Fig. 1B), which was defined as DeepVS model.

### Virtual screening of anti-cancer natural compounds based on DeepVS

We then utilized DeepVS for the screening of anti-cancer active ingredients, selecting compounds with a prediction probability greater than 0.5 as potential anti-cancer agents. From the TCMID candidate library (containing 25,918 compounds) and a small natural product candidate library (2,042 compounds), we identified 1,431 and 423 potential anti-cancer compounds, respectively. After removing duplicates, a total of 1,578 active compounds were retained. These were compiled into a potential candidate drug library for anti-cancer bioactivity and used to screen for LOXL2 inhibitors.

Subsequently, based on the molecular features of compounds extracted by the DeepVS model, we employed a dimensionality reduction algorithm to explore the chemical space of these potential inhibitors. Molecular features of the entire screened compound set were extracted from the last fully connected layer of the GCN model, encoding the molecular information into a 256-dimensional vector.

**Fig. 2:**
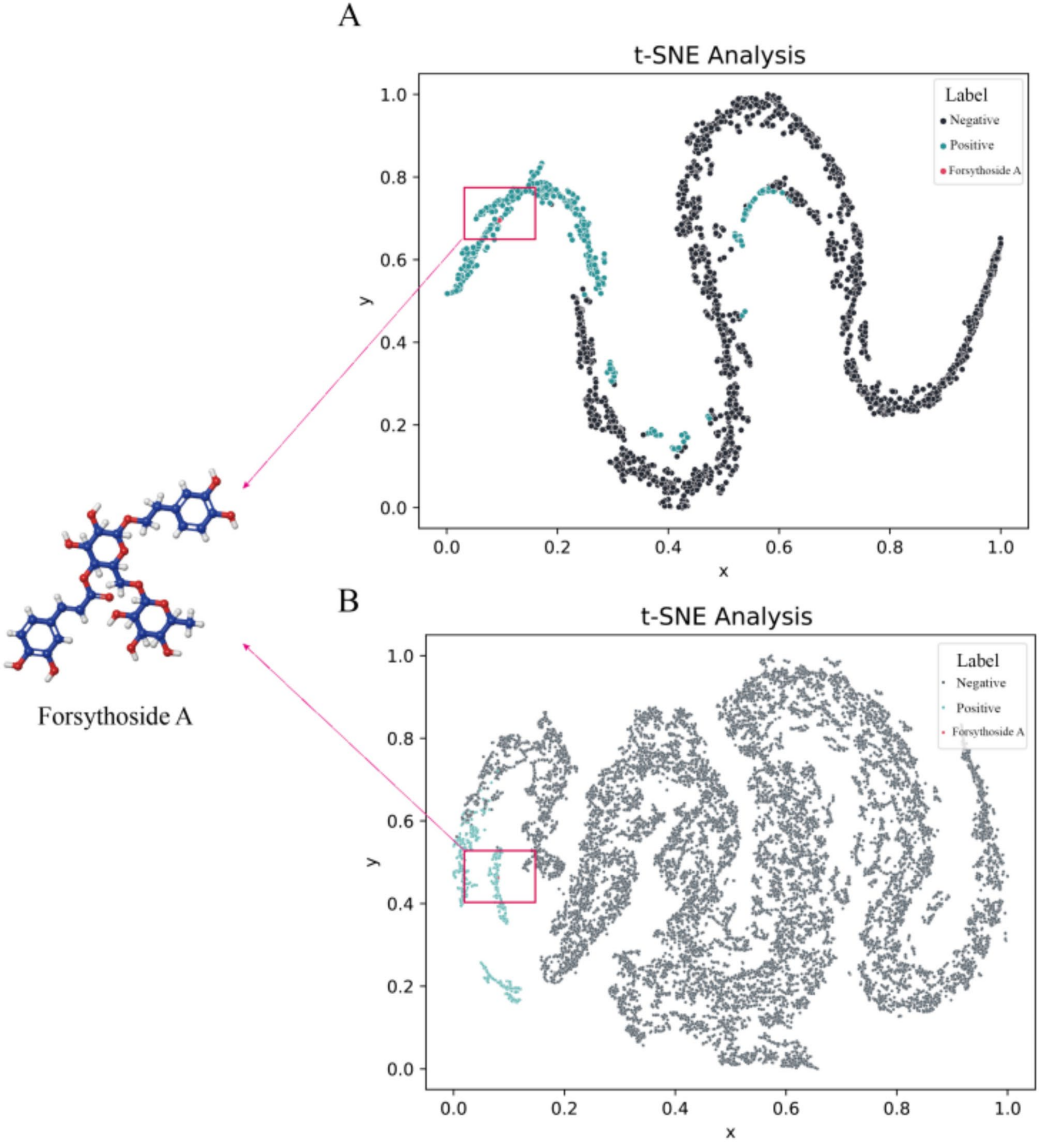
The t-SNE analysis of predicted active and inactive compounds following DeepVS screening of natural product libraries. (**A**) Screening results from the TCMID traditional Chinese medicine compound library using DeepVS (Forsythiaside A is highlighted within the red box); (**B**) Screening results from a smaller natural product library using DeepVS. The area designated for Forsythiaside A is indicated within the red box.

The t-SNE method was then applied to analyze and visualize the potential chemical space of the model predictions. The results revealed that the active (marked in green) and inactive (marked in black) components predicted by DeepVS exhibited a clear hierarchical clustering trend (Fig. 2A and B). The t-SNE outcomes indicated that the model possesses commendable feature extraction and representation capabilities. Moreover, within the red-boxed area of the figure, a natural compound of subsequent focus, Forsythoside A, was also marked in red.

### LOXL2 inhibitors discovery based on molecular Docking

We performed flexible molecular docking of the compounds from a natural product candidate library with LOXL2. Ligand preparation was performed on the molecular structure of 1454 natural products screened by DeepVS. By applying Protein Preparation and Receptor Grid Generation module, LOXL2 was deemed suitable for molecular docking. After eliminating the overlapping molecules, 1792 specific molecular docking results were obtained for LOXL2. For docking results, docking score and specific docking poses were used as the docking metrics. Analogous to energy, favourable scores were negative, and the lower the score (more negative), the better the affinity. Therefore, lower Glide Gscores indicated better ligand-protein binding affinity. The docking scores of LOXL2 inhibitor, lenumlostat, were employed as a control to screen the potential active inhibitor of LOXL2.

**Fig. 3:**
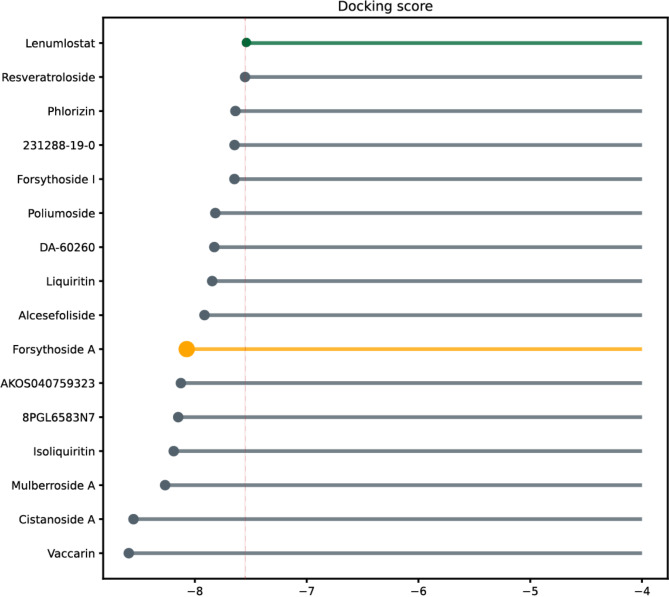
Top 30 results of molecular docking scores between compounds from the natural product candidate library and LOXL2.

Based on the molecular docking results, 15 of these natural compounds exhibited lower docking scores compared to the LOXL2 inhibitor, as shown in Fig. 3. Combining the deep learning and molecular docking results, we ultimately identified these 15 compounds as potential LOXL2 inhibitors, as summarized in Table 1.

**Table 1:** Potential LOXL2 inhibitors and their anti-cancer activities predicted by DeepVS.

PubChem CID	Compounds	DeepVS prediction	Docking score
71307582	Vaccarin	Positive	-8.591
71307448	Cistanoside A	Positive	-8.549
6443484	Mulberroside A	Positive	-8.266
5318591	Isoliquiritin	Positive	-8.189
10169367	8PGL6583N7	Positive	-8.149
10146542	AKOS040759323	Positive	-8.125
5281773	Forsythoside A	Positive	-8.073
11828754	Alcesefoliside	Positive	-7.914
503737	Liquiritin	Positive	-7.845
134715163	DA-60260	Positive	-7.826
6442411	Poliumoside	Positive	-7.817
23958169	Forsythoside I	Positive	-7.646
124222341	231288-19-0	Positive	-7.645
6072	Phlorizin	Positive	-7.637
5322089	Resveratroloside	Positive	-7.551
122536283	Lenumlostat	Positive	-7.544
66570668	(2-chloropyridin-4-yl) methylamine hydrochloride	Positive	-6.071

### Preliminary identification and pharmacological analysis of Forsythoside A

Preliminary, graph convolutional network method (DeepVS) was taken as the primary virtual screening method, profiting by its robust performance of parameter optimization and feature extraction. DeepVS shows superior performance for LOXL2 and cancer related inhibitor prediction, which achieved mean 0.942 AUC score.

It was carried out to determine the bioactive profile of Forsythoside A as potent LOXL2 inhibitor, out of 25,918 TCM ingredient databases and 2,042 natural product libraries. A comprehensive review of the literature indicates that Forsythiaside A exhibits a broad spectrum of biological activities, including immunomodulatory, anti-inflammatory^21^, antiviral^22^, antioxidant, and anti-endotoxin effects^23^. From the chemical informatics prediction space constructed by the deep learning model, we can observe the position of Forsythoside A (as shown in Fig. 3), which is located in the main positive predictive clusters.

**Fig. 4:**
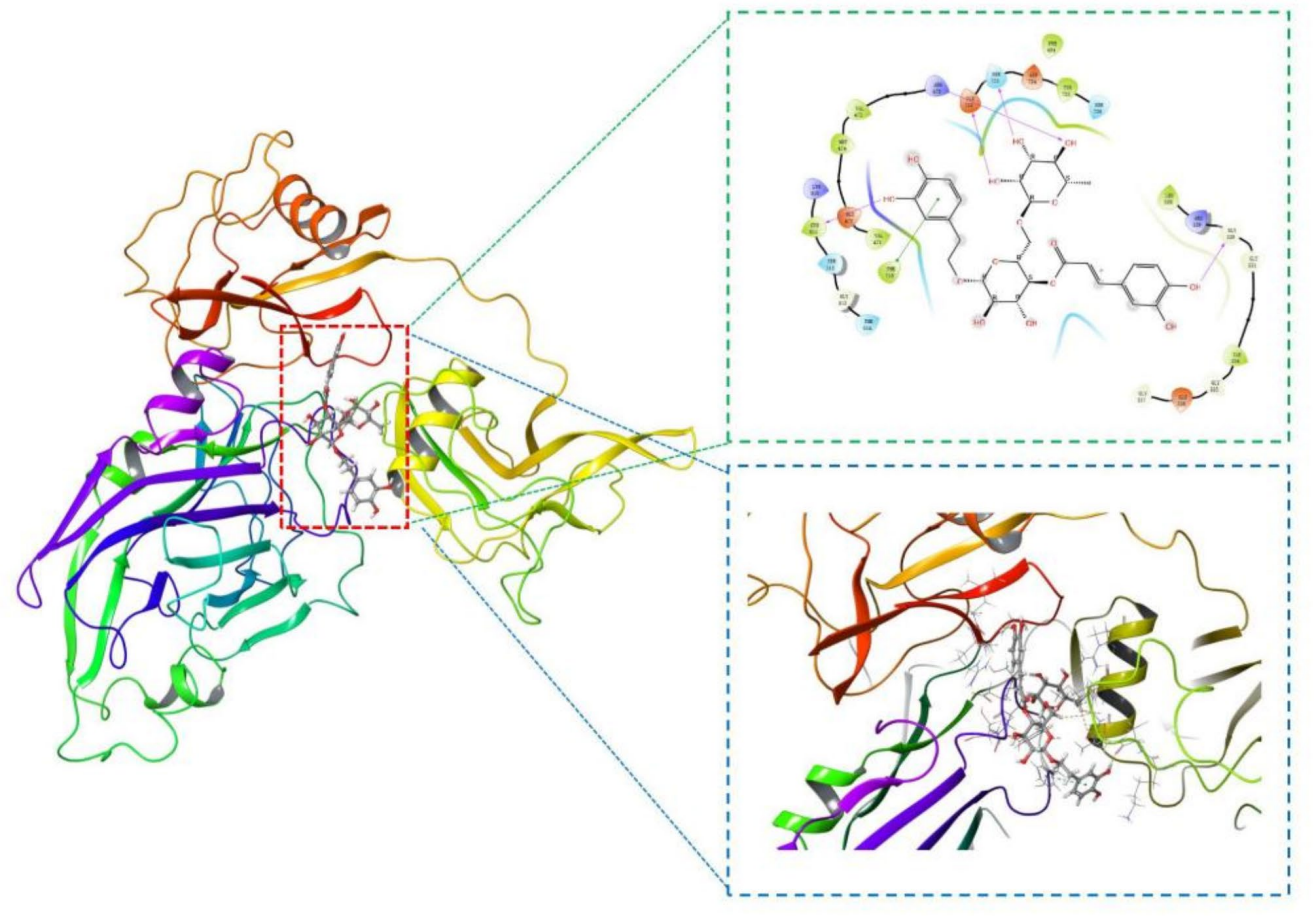
Molecular docking analysis reveals the specific binding mode and affinity of Forsythoside A with LOXL2.

**Fig. 5:**
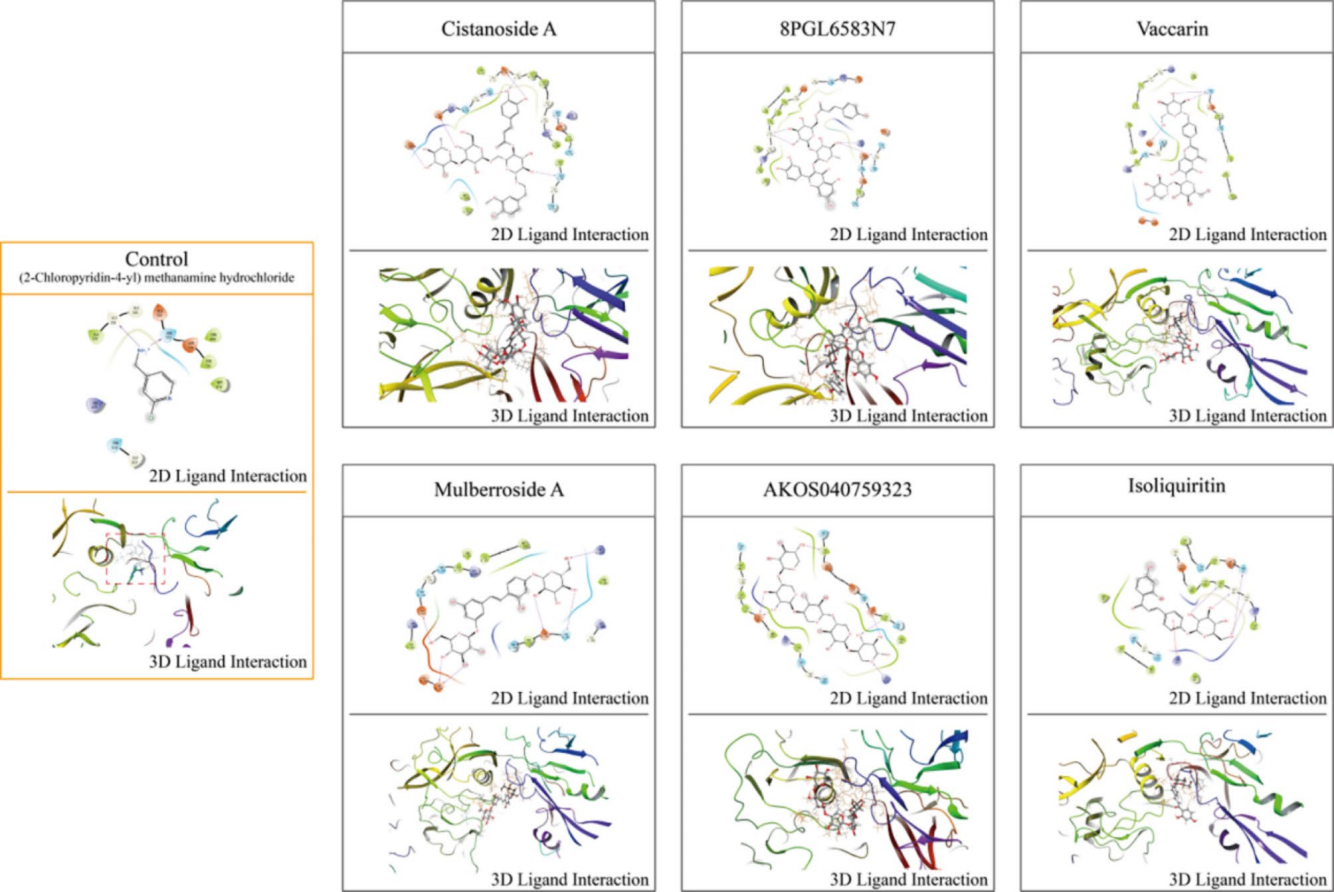
The specific binding mode of control group and other top six compounds with LOXL2.

Subsequently, molecular docking was performed to identify the specific binding mode and affinity of Forsythoside A with LOXL2. The GScore was introduced as one of binding affinity metrics, and Forsythoside A achieved a binding affinity of -8.073 kal/mol, indicating a superior binding conformation with LOXL2. The (2-chloropyridin-4-yl) methylamine hydrochloride, serving as the control group, exhibited a docking score of -6.071 kal/mol.The interactions were dominated in the region of Gly 330, Gly 331, Gly 335, Glu 336, CYS 511 and SER 723 amino acid residues content due to pronouncing existence of active sites in the region (Fig. 4). Additionally, molecular docking interaction poses for control drug and six top compounds are presented in Fig. 5.

### Molecular dynamics simulation of protein-ligand complexes for the identification of potent LOXL2 inhibitors

#### Comparative analysis of complex stability and conformational dynamics through root mean square deviation (RMSD)

**Fig. 6:**
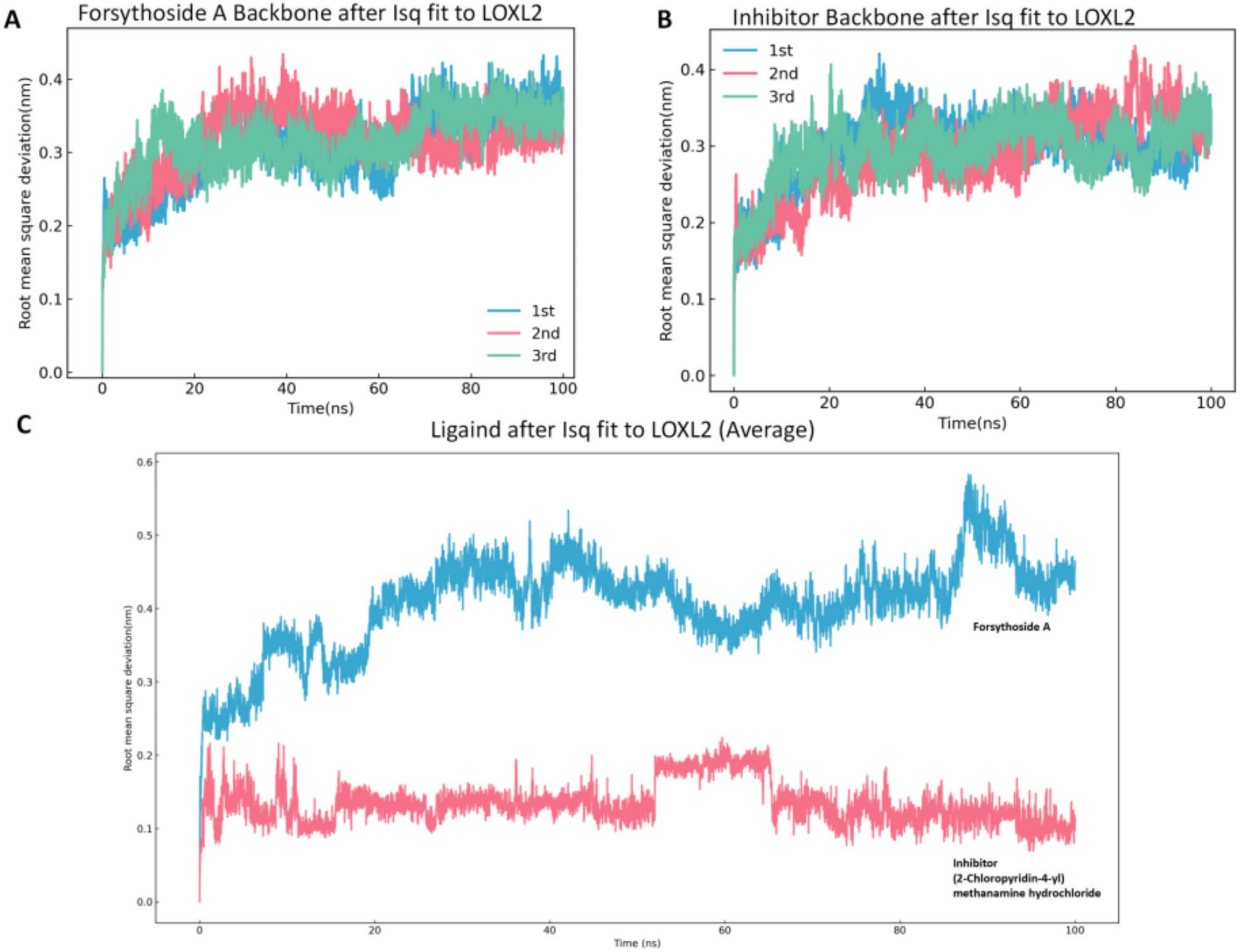
Molecular dynamics RMSD profiles of the complexes. (**A**) Parallel RMSD trajectories of triplicate simulations for the FA-LOXL2 complex. (**B**) Parallel RMSD trajectories of triplicate simulations for the inhibitor-LOXL2 complex. (**C**) Comparative analysis of average RMSD values for Forsythoside A (blue) and inhibitor (red) complexes aligned to LOXL2.

RMSD analysis was conducted to assess the conformational deviations of protein-ligand complexes relative to their initial structures throughout the simulation. Elevated RMSD values signify increased structural plasticity over time. Figure 6 displays the molecular dynamics simulation outcomes of Forsythoside A and LOXL2-inhibitor (2-chloropyridin-4-yl) methylamine hydrochloride in complex with LOXL2, evaluated via RMSD metrics. Panels A and B depict the RMSD trajectories of the FA-LOXL2 and inhibitor-LOXL2 complexes, respectively, with each curve representing an independent replicate. Both systems demonstrated stable convergence, indicative of dynamic equilibrium attainment within the 100-ns simulation period.

Specifically, the RMSD curves of the FA-LOXL2 complex (Fig. 6A) oscillated within a narrow range of 0.2–0.4 nm, lacking sustained upward trends. The inhibitor-LOXL2 complex (Fig. 6B) exhibited comparable stability, with RMSD values remaining below 0.3 nm throughout the simulations. This consistency across triplicate simulations corroborates the structural integrity of both complexes.

Figure 6C presents a comparative analysis of the average RMSD trajectories for the Forsythoside A and inhibitor complexes aligned to LOXL2. The FA-LOXL2 complex (blue curve) consistently exhibited higher average RMSD values than the inhibitor complex (red curve), suggesting enhanced conformational flexibility. This increased structural adaptability may align with the multi-target pharmacological mechanisms of traditional medicines, which often exert therapeutic effects through synergistic interactions with multiple binding sites.

In summary, RMSD analysis revealed stable equilibration for both complexes during the 100-ns simulations. However, the FA-LOXL2 system displayed greater conformational variability compared to the inhibitor-LOXL2 complex, potentially reflecting its polypharmacological nature. These findings provide valuable insights into the dynamic behavior of FA-LOXL2 interactions and lay a foundation for further mechanistic studies.

#### Binding free energy analysis of Forsythoside A and inhibitor complexes with LOXL2

**Fig. 7:**
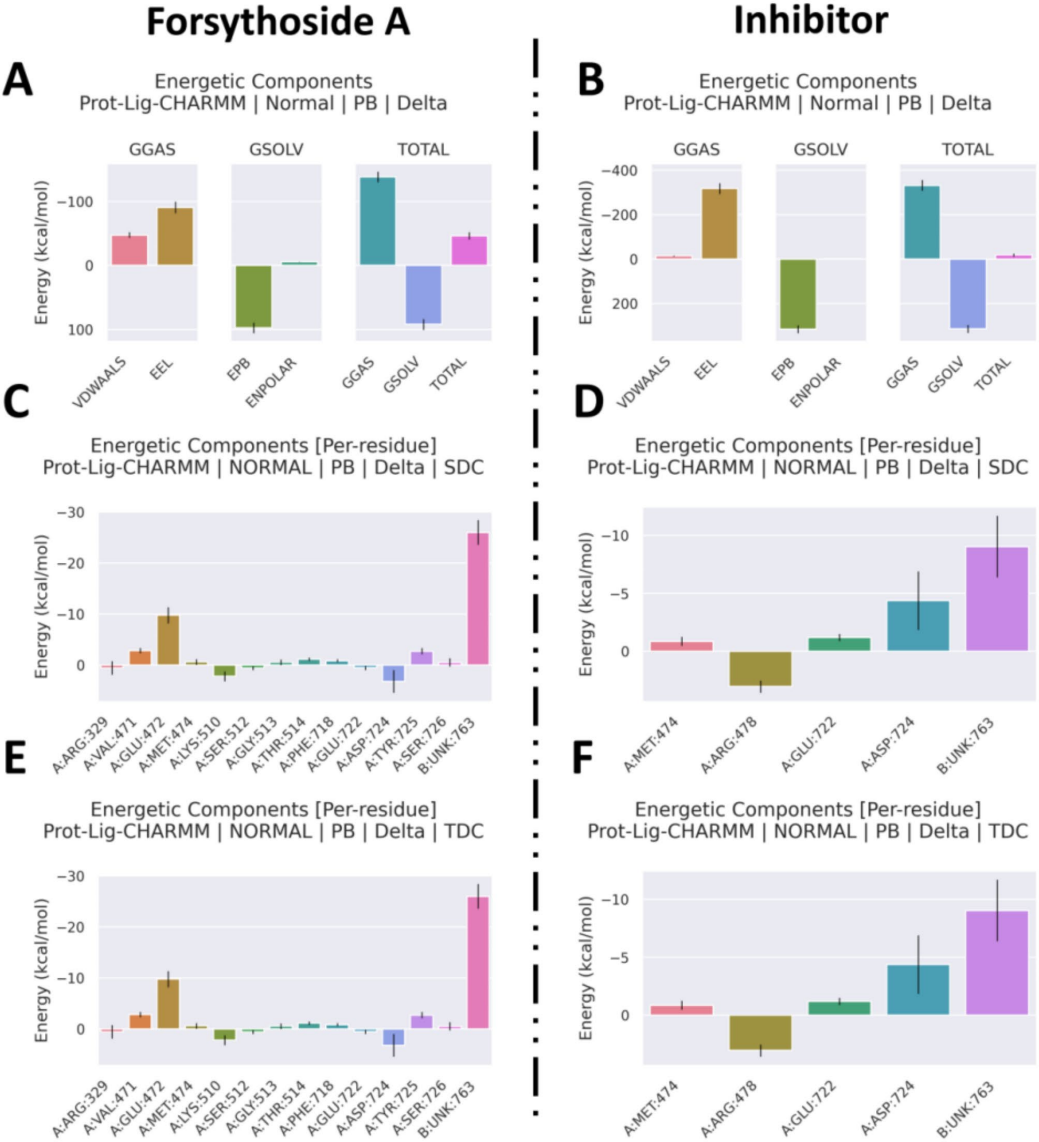
Binding free energy analysis of Forsythoside A and inhibitor with LOXL2. (**A**, **B**) Energy property decomposition profile of the Forsythoside A and Inhibitor. (**C**, **D**) Amino acid energy decomposition profile of the Forsythoside A and Inhibitor molecule (SDC model). (**E**, **F**) Amino acid energy decomposition profile of the Forsythoside A and inhibitor (TDC model).

The Molecular Mechanics/Poisson-Boltzmann Surface Area (MM/PBSA) method was employed to evaluate the binding free energy of small molecules with their respective protein targets. More stable complexes exhibit lower binding free energy. As illustrated in Fig. 7; Table 2, the Forsythoside A complex with LOXL2 demonstrated greater stability, as indicated by the total energy change (ΔTOTAL): -46.36 ± 7.02 kcal/mol for Forsythoside A versus − 18.85 ± 17.88 kcal/mol for the inhibitor.

**Table 2:** Binding energy results for Forsythoside A and inhibitor complexes with LOXL2.

Delta energy (kcal/mol)*	Forsythoside A**	(2-chloropyridin-4-yl)methylamine hydrochloride**
ΔBOND	0 ± 2.72	0 ± 1.33
ΔANGLE	0 ± 4.79	0 ± 1.64
ΔDIHED	0 ± 2.49	0 ± 1,39
ΔVDWAALS	-47.71 ± 0.39	-14.4 ± 0.14
ΔEEL	-90.92 ± 5.89	-318.44 ± 13.47
Δ1-4VDW	0 ± 1.74	0 ± 0.52
Δ1-4EEL	0 ± 1.81	0 ± 0.61
ΔEPB	97.79 ± 3.80	315.99 ± 11.76
ΔENPOLAR	-5.52 ± 0.05	-2.01 ± 0.01
ΔEDISPER	0 ± 0.00	0 ± 0.00
ΔGGAS	-138.63 ± 5.90	-332.83 ± 13.47
ΔGSOLV	92.27 ± 3.81	313.98 ± 11.76
ΔTOTAL	-46.36 ± 7.02	-18.85 ± 17.88

*Energy component. **Average ± Standard deviation.

In terms of van der Waals interactions (ΔVDWAALS), Forsythoside A exhibited a significantly lower value (-47.71 ± 0.39 kcal/mol) than the inhibitor (-14.4 ± 0.14 kcal/mol), suggesting stronger van der Waals forces contributing to binding stability. For polar solvation energy (ΔEPB), the Forsythoside A complex demonstrated a value of 97.79 ± 3.80 kcal/mol, whereas the inhibitor displayed a markedly higher value of 315.99 ± 11.76 kcal/mol, indicating greater solvation energy changes during binding, potentially due to stronger solvent interactions.

Considering all energy components, the Forsythoside A complex exhibited superior binding stability, likely attributed to favorable binding characteristics across multiple energy terms, including enhanced polar solvation energy, surface energy, and solvation free energy, alongside reduced non-polar solvation energy.

#### Free energy landscape (FEL) analysis

**Fig. 8:**
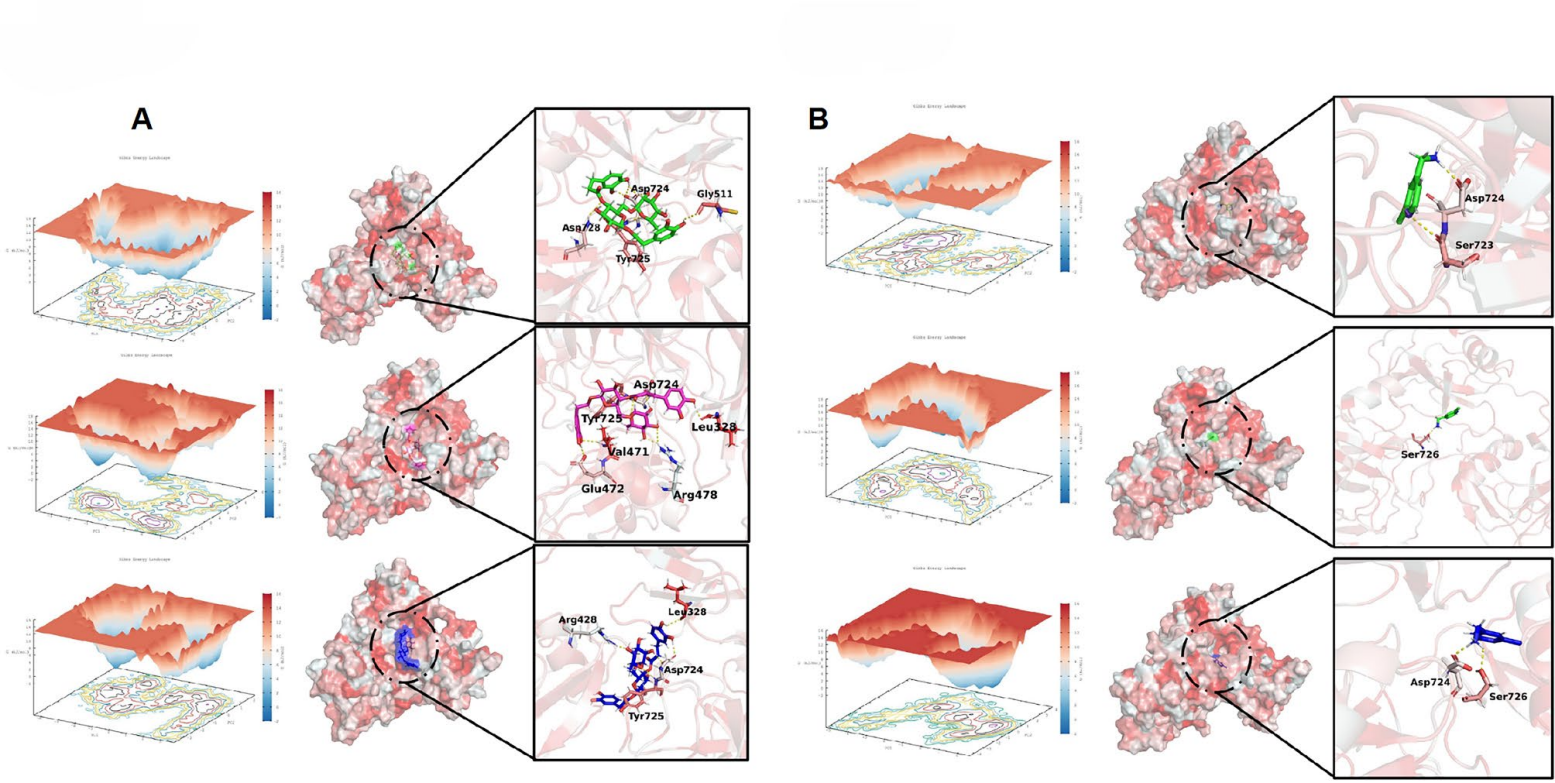
Free Energy Landscape (FEL) analysis, minimum-energy conformations, and hydrogen bond interactions for the FA-LOXL2 complex (**A**) and inhibitor-LOXL2 complex (**B**).

Free Energy Landscape (FEL) analysis is a powerful tool for evaluating energy variations in molecular interactions, providing insights into the thermodynamic properties of ligand-protein binding. On the left side of Fig. 8A, FELs from three independent simulations are displayed, with color gradients ranging from blue (low energy) to red (high energy). The system predominantly transitions toward lower energy states, indicating thermodynamically favorable interactions between Forsythoside A and LOXL2. On the right side of Fig. 8A, the lowest-energy conformations from each simulation are highlighted, revealing specific hydrogen bond interactions with key amino acid residues (e.g., Asp724, Glu722, and Arg478), which stabilize the FA-LOXL2 complex.

Similarly, Fig. 8B presents the FEL and stable conformations for the inhibitor-LOXL2 complex. Key residues involved in inhibitor binding include Asp724, Ser723, and Ser726. Compared to the Forsythoside A results, the FA-LOXL2 complex (Fig. 8A) exhibited a broader energy distribution, suggesting more diverse interactions. Additionally, the FA-LOXL2 complex formed a more extensive hydrogen bond network involving a greater number of amino acid residues, contributing to its enhanced binding stability.

In summary, while the inhibitor-LOXL2 interaction is relatively straightforward, the FA-LOXL2 interaction is more complex and diversified, characterized by a broader hydrogen bond network. This complexity may underlie the superior binding stability of Forsythoside A. These findings provide valuable insights into the mechanisms of action of Forsythoside A and the inhibitor, offering critical guidance for drug design.

### Experimental validation of Forsythoside A

**Fig. 9:**
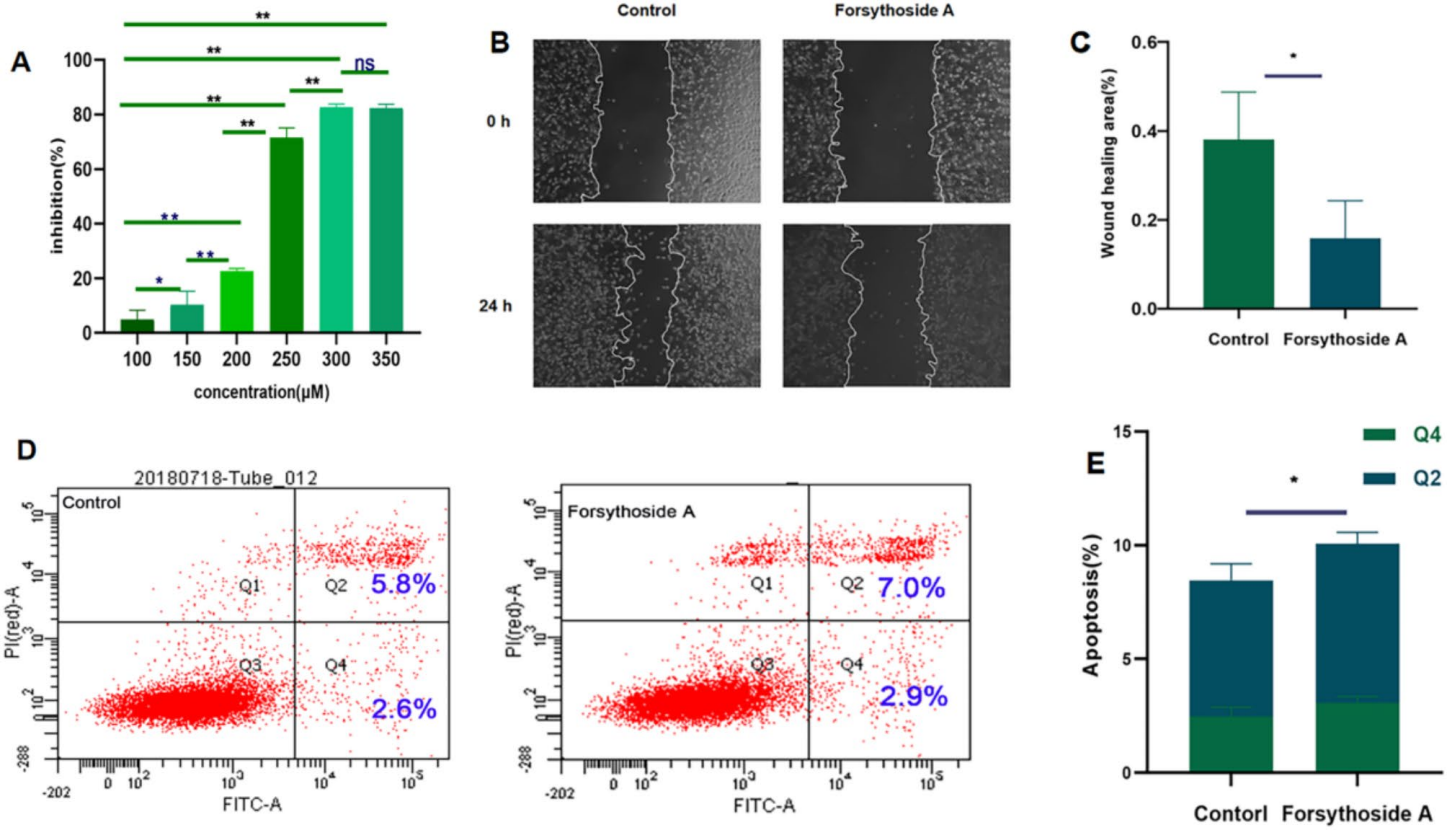
Effect of Forsythoside A on the biological behavior of cancer cells. (**A**) CCK-8 assay was used to verify the effect of Forsythoside A on cell proliferation; (**B**, **C**) The effect of Forsythoside A on cell migration ability was examined by comparing the cell scratch assay at time 0 with that at time 24; (**D**, **E**) Flow cytometry was used to examine the effect of Forsythoside A on apoptotic ability of the cells. *n* = 3. *P < 0.05, **P < < 0.01.

We used CT26 cells as experimental material to verify the effect of Forsythoside A on cellular behavior in carcinogenesis and progression in vitro. Results showed that Forsythoside A can significantly inhibit cell proliferation in a dose-dependent manner (Fig. 9A), with an IC50 value of 230 µM, 50 µM was selected as the concentration of Forsythoside A administration in the subsequent experiments. To further verify the inhibition of tumour migration ability and the ability of Forsythoside A to promote tumour apoptosis, we used 50 µM Forsythoside A on CT26 cells after its treatment. It was found that the migration of tumour cells was weakened and the migration ability was inhibited after the addition of Forsythoside A compared with control cells (Fig. 9B and C), and the results of flow-through showed that the number of apoptotic cells was increased after the administration of Forsythoside A, and that the apoptosis was promoted by Forsythoside A (Fig. 9D and E).

**Fig. 10:**
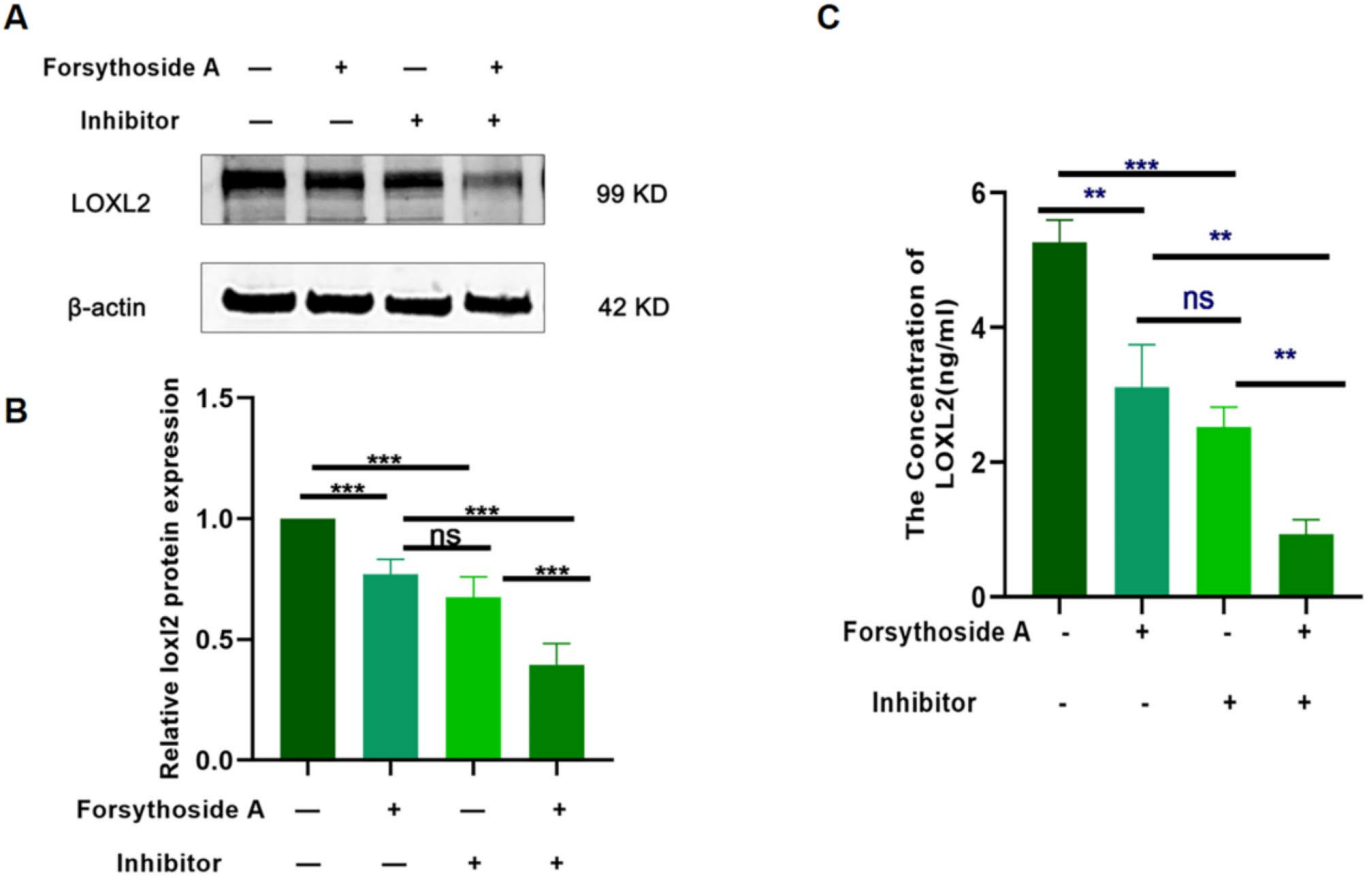
Effect of Forsythoside A on LOXL2 protein expression. (**A**, **B**) Western blotting assays examined the LOXL2 protein expression levels. (**C**) ELISA detected the amount of LOXL2 expression in cell Each experiment was performed in triplicate. (The results have been cropped.) *n* = 3. *P < 0.05, **P < 0.01, ***P < 0.001.

To observe the inhibitory effect of Forsythoside A on LOXL2, the LOXL2 inhibitor (2-Chloropyridin-4-yl) methanamine hydrochloride was used as a control, and the expression level of LOXL2 was detected by western blotting. Results showed that the expression of LOXL2 was lower in the cells treated with Forsythoside A alone or with the inhibitor (administered at a concentration of 63 nmol/L) as compared with the control group. However, the inhibitory effect of forsythoside A was lower than that of the control group (2-chloropyridin-4-yl) methylamine hydrochloride. LOXL2 expression was significantly decreased in the group treated with both Forsythoside A and inhibitor compared with the group treated with Forsythoside A or inhibitor (Fig. 10A and B).The supernatants of cells treated with different treatments were also collected for ELISA (Fig. 10C). The results showed that the expression of LOXL2 was reduced in cells treated with forsythoside A alone or with the inhibitor compared to that in the control. Similarly, the inhibitory effect of forsythoside A was lower than that of the control (2-chloropyridin-4-yl) methylamine hydrochloride).The complete Western blotting results are shown in Fig. S1.

## Discussion

In our study, deep learning method was taken as the primary virtual screening method, profiting by its robust performance from parameter optimization and feature extraction^24^. It has enhanced capabilities in feature representation and generalisation, which improves the efficiency of large-scale application of data and makes large-sample training and application possible^25^. Combining it with molecular docking further improves the success of drug discovery, and with the algorithms and software continue to be upgraded, computer-aided drug discovery is focusing on the use of structure-based approaches, and deep learning is playing an increasingly important role in early drug discovery areas such as virtual screening and molecular docking.

LOXL2 has been shown to have an important role in certain fibrotic diseases and cancers, and selective inhibition of LOXL2 has received extensive research attention for its potential therapeutic advantages in treating these diseases. Consistent with contemporary trends in drug discovery, virtual screening combined with artificial intelligence has become a widely accepted approach due to its cost-effectiveness and efficiency. Therefore, we incorporated this innovative approach into our study.

In the initial phase, we optimized a deep learning framework based on the graph convolutional network approach, DeepVS, conducted a screening of a comprehensive database of Chinese medicine ingredients and a limited database of natural products, and subsequently predicted the potential inhibitor of LOXL2, which was identified as the natural compound Forsythoside A.

Subsequently, molecular docking of the screened compounds was conducted. Through the application of protein preparation and receptor lattice generation modules, LOXL2 was determined to be suitable for molecular docking. The docking score of the LOXL2 inhibitor was utilized as a control to screen LOXL2 as a potential active inhibitor. The results indicated that the natural product Forsythoside A exhibited slightly better docking results for LOXL2 compared to LOXL2 inhibitors. Forsythoside A possesses remarkable anti-inflammatory and antioxidant properties, as well as potent therapeutic effects in cancer^23^. Consequently, it can be concluded that the natural product Forsythoside A may function as a potent inhibitor of LOXL2.

Forsythoside A is derived from Shuang-Huang-Lian, a well-known traditional Chinese medicine formulation with documented pharmacological activities^26^. Forsythoside A has been extensively studied and reported in literature for its antifibrotic properties. It has been shown to regulate pulmonary fibrosis by inhibiting endothelial-to-mesenchymal transition and lung fibroblast proliferation via the PTPRB signaling pathway^27^. Additionally, it alleviates high glucose-induced oxidative stress and inflammation in podocytes by inactivating MAPK signaling through MMP12 inhibition^28^, and the anticancer effect of Forsythoside A in esophageal squamous cell carcinoma was approved^29^, which highlights its potential therapeutic value beyond just LOXL2 inhibition. In addition there have been articles reporting that Forsythoside A’s pharmacokinetic properties, including absorption enhancers based on tight junctions, have been explored in relation to its antiviral activity^26^, providing a foundation for understanding its potential in vivo behavior. Therefore, the choice was made to validate the therapeutic potential of forsythoside A in the context of LOXL2 inhibition, leveraging its known biological activities and established research background. Although other compounds may have shown marginally better docking scores, the comprehensive biological profile, established mechanisms, and prior research on forsythoside A made it the most suitable choice for our study.

To further confirm this conclusion, we selected CT26 cells for in vitro experiments, which showed that Forsythoside A could inhibit cell proliferation, reduce cell migration, and promote cell apoptosis. Protein experiments also showed the same trend. Thus, we further confirmed that the natural product Forsythoside A is an inhibitor of LOXL2, while the efficiency and accuracy of drug discovery were improved by utilizing techniques such as deep learning molecular docking. However, we only derived that Forsythoside A can inhibit the expression level of LOXL2, and its specific regulatory mechanism is still unclear, and we may continue to further investigate its specific occurrence mechanism through multi-omic approaches, deep learning, and other methods in the future.

## Electronic supplementary material

Below is the link to the electronic supplementary material.

Supplementary Material 1

## Data Availability

The datasets generated during and/or analysed during the current study are available from the corresponding author on reasonable request.
