# Supplementary material for: Artificial intelligence accelerates the identification of nature-derived potent LOXL2 inhibitors
Source: Sci Rep. 2025 Mar 27;15:10540. doi: 10.1038/s41598-025-95530-9 (PMC11950171; doi:10.1038/s41598-025-95530-9)
Supplement: Supplementary file 1 — Supplementary Material 1 [file 41598_2025_95530_MOESM1_ESM.pdf]

Figure S1

Repeat 1 and 2

LOXL2 99 kd (From right to left)

A **B**

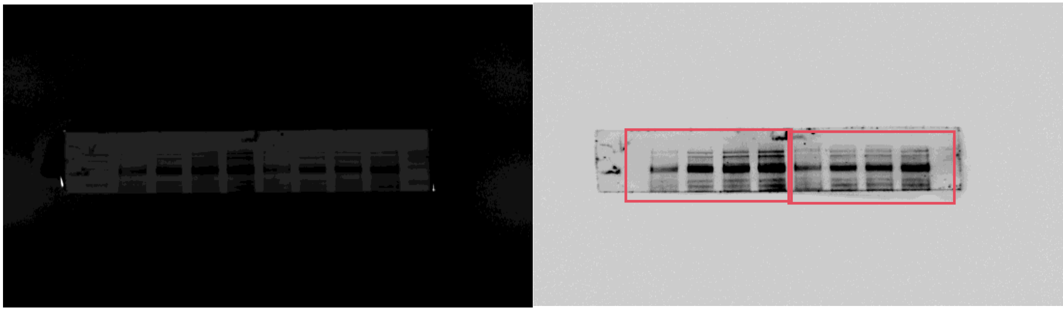

Forsythoside A	+	—	+	—	+	—	+	—
Inhibitor	+	+	—	—	+	+	—	—

β -actin 42 kd

A **B**

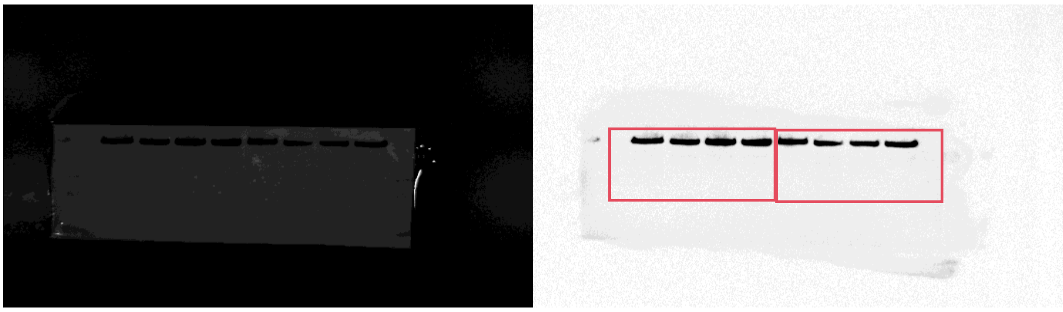

C

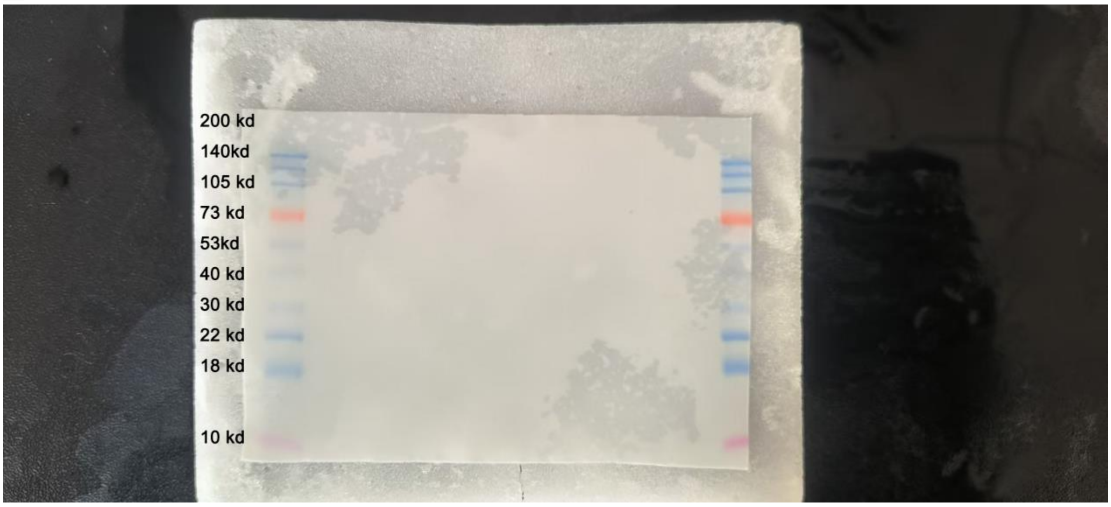

D

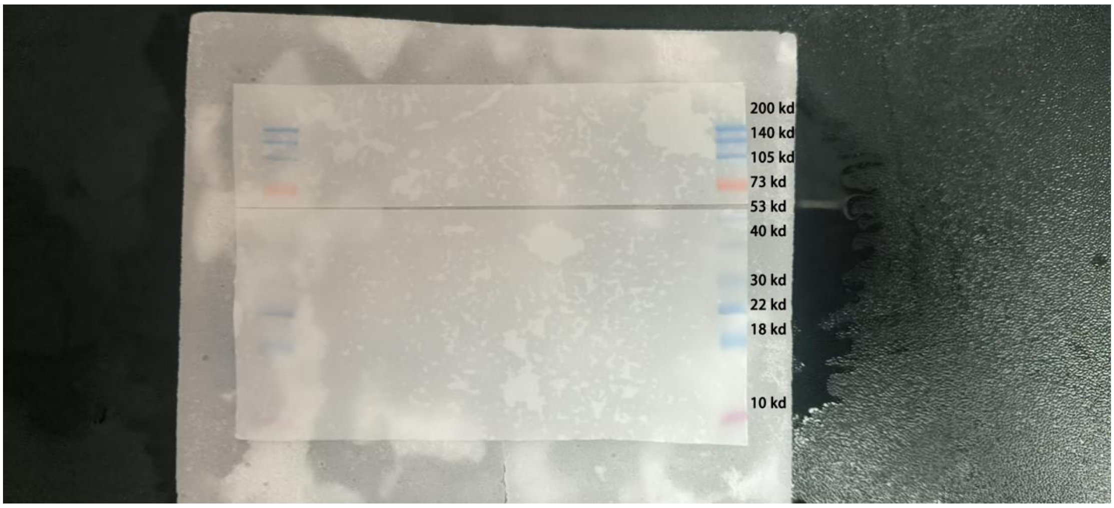
200 kd
140 kd
105 kd
73 kd
53 kd
40 kd
30 kd
22 kd
18 kd
10 kd

E

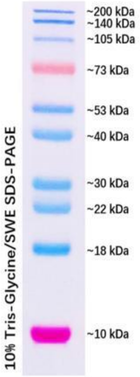
~200 kDa
~140 kDa
~105 kDa
~73 kDa
~53 kDa
~40 kDa
~30 kDa
~22 kDa
~18 kDa
~10 kDa
10% Tris-Glycine/SWE SDS-PAGE

Repeat 3

LOXL2 99 kd

A

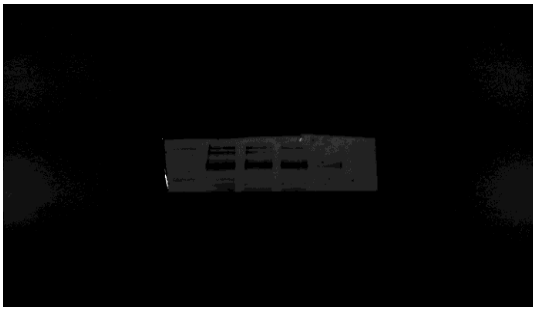

B (From left to right)

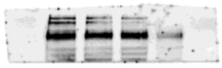

Forsythoside A — + — +

Inhibitor — — + +

β -actin 42 kd

A

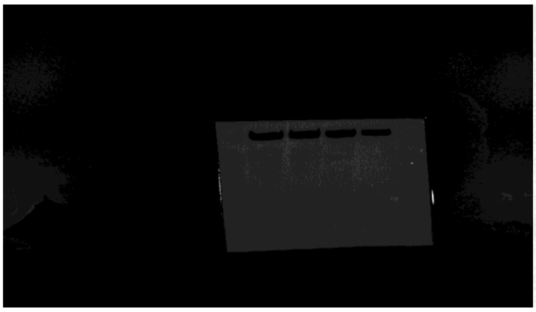

B

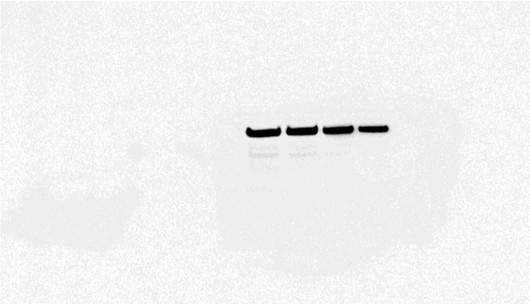

C

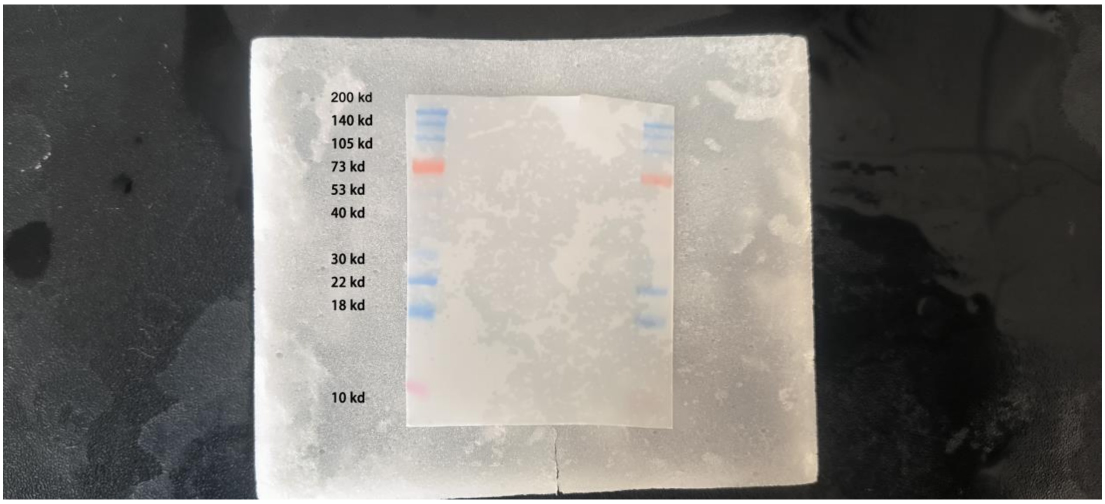

D

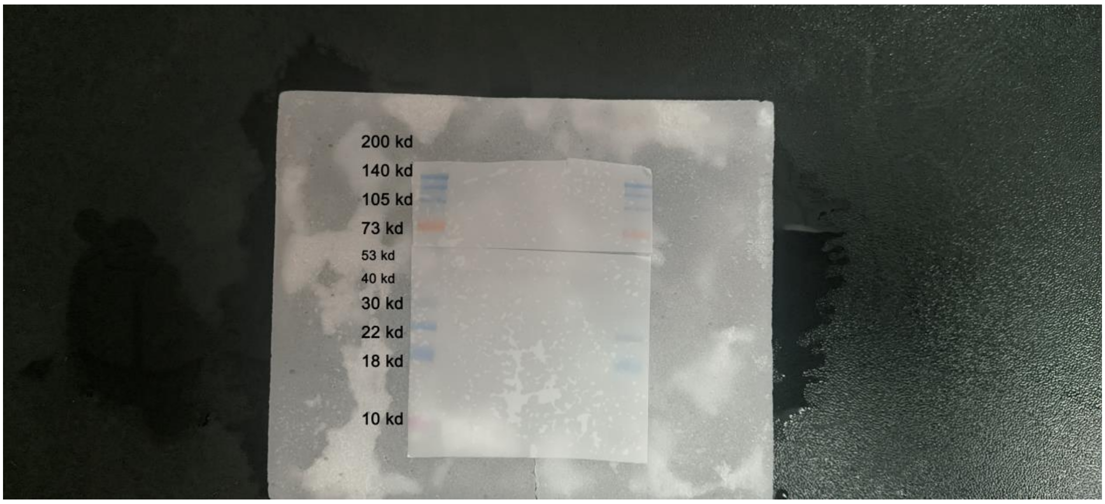

Complete three-time Western blot image in Fig. 10. A is the strip map with maker, B is the sample map, C is the original whole film and D is the whole film after cropping and piecing together and E is the maker used.
